# Learning and Recall of Medical Treatment-Related Information in Older Adults Using the Differential Outcomes Procedure

**DOI:** 10.3389/fpsyg.2018.00157

**Published:** 2018-02-14

**Authors:** Victoria Plaza, Michael Molina, Luis J. Fuentes, Angeles F. Estévez

**Affiliations:** ^1^Department of Psychology, Autonomous University of Chile, Santiago, Chile; ^2^Department of Basic Psychology, Autonomous University of Madrid, Madrid, Spain; ^3^Department of Education, Mayor University, Santiago, Chile; ^4^Department of Basic Psychology and Methodology, University of Murcia, Murcia, Spain; ^5^CERNEP Research Center, University of Almería, Almería, Spain; ^6^Department of Psychology, University of Almería, Almería, Spain

**Keywords:** adherence to treatment, aging, differential outcomes procedure, learning, long-term memory

## Abstract

It has recently been reported that the differential outcomes procedure (DOP) might be one of the therapeutical techniques focused at promoting autonomy in the elderly to deal with their medical issues. Molina et al. (2015) found that a group of healthy young adults improved their learning and long-term retention of six disorder/pill associations when each relationship to be learned was associated with a particular reinforcer (the differential outcomes condition) compared to when they were randomly administered (the non-differential outcomes condition). In the present study, we extend these findings to older adults who usually show difficulties to remember to take their medications as prescribed. Participants were asked to learn the association between three pills and the specific time at the day when they had to take each medication. Two memory tests were also performed 1 h and 1 week after completing the training phase. Results showed a faster learning of the task and long-term retention of the previously learned associations (pill/time of day) when differential outcomes were used. Furthermore, the older adults’ performance in the learning and memory phases did not differ from that of the younger adults in the DOP condition. These findings demonstrate that this procedure can help elderly people to ameliorate not only their learning, but also their long-term memory difficulties, suggesting the potential for the DOP to promote adherence to treatment in this population.

## Introduction

Does the way in which the outcomes are administered after each correct response shape learning and memory processes? This is a crucial question that has been partially addressed by research investigating the effect of using the differential outcomes procedure (DOP; Trapold, 1970) in tasks of discriminative learning. The DOP consists of assigning a specific and unique consequence to every stimulus-response association that has to be learned. Let us explain the procedure through the following example: imagine grandpa has severe problems to recall the names of his two grandchildren (grandchild A and grandchild B) and associate them with their correct names (name A and name B). The training with differential outcomes consists of pairing one specific outcome, in this case a reinforcer, with each stimulus (grandchild) – response (to correctly say his or her name) association (e.g., a kiss when grandchild A is associated correctly with name A, and a hug when grandchild B is correctly associated with name B). When applying this procedure learning is more effective and faster than when outcomes are applied randomly, an effect which is known as the Differential Outcomes Effect (Trapold and Overmier, 1972).

Even though first data about this effect comes from studies on animals (for revision see, Urcuioli, 2005), during the last years the number of investigations on the potential benefits of the DOP for humans has risen significantly. More precisely, it was demonstrated that the DOP can be beneficial for the improvement of discriminative learning in populations with and without pathology, for children (e.g., Maki et al., 1995; Estévez et al., 2001, 2003, 2016; Martínez et al., 2009, 2012, 2013) as well as for adults (e.g., Malanga and Poling, 1992; Joseph et al., 1997; Miller et al., 2002; Easton, 2004; Estévez et al., 2007; Mok and Overmier, 2007).

Furthermore, the procedure improves not only discriminative learning but also working memory when each to-be-remembered stimulus is associated with a specific outcome in different population groups (e.g., Plaza et al., 2011, 2013; Martella et al., 2012; Esteban et al., 2014a,b, 2015), especially in older adults without cognitive disturbances (López-Crespo et al., 2009), patients with alcohol dementia (Hochhalter et al., 2000), and patients with Alzheimer’s disease (Plaza et al., 2012). In these last investigations, improved delayed face recognition was observed when differential outcomes were applied.

Recently, Molina et al. (2015) went further to explore whether the DOP could improve handling medication prescriptions in a group of healthy young adults. More precisely, the researchers simulated a situation where they “prescribed” participants a treatment consisting of six different drugs (or pills). Participants had to learn to associate each pill with the pathology it was prescribed for. Furthermore, to evaluate long-term effects of the differential outcomes, two memory tests were performed, 1 h and 1 week after completing the learning phase. The findings showed that the application of the DOP enhanced learning and long-term memory of the previously learned associations. As the authors pointed out, this would suggest it may be a suitable technique for intervention programs aimed at improving memory conditions that foster adherence to treatment; especially in populations with learning and memory difficulties, such as older adults or patients with neurodegenerative disorders like Alzheimer’s or Parkinson’s disease.

The difficulty to learn and retain relevant aspects of medical treatment is a public health concern worldwide, which implies an unjustified increase in the cost of health systems and has negative effects on patient’s health, such as increased hospital admissions, worsening of the disease and even death (Chisholm-Burns and Spivey, 2003). Some researchers have reported serious difficulties of older adults to handle critical aspects related to their diseases (Stewart and Caranasos, 1989), mainly related to the complexity of medical treatments. This makes older adults particularly vulnerable since normal aging usually accompanies a decline in cognitive processes that are necessary for patients to retain medical indications. The seemingly simple task to remember to take the prescribed medication requires, among others, a properly operating working memory, long-term memory, and prospective memory (Park, 1992), which are usually affected by normal aging (Hedden and Gabrieli, 2004). Therefore, the application of methods targeted to improve memory of medical prescription, as well as discrimination between different drugs or medication routines would be very helpful about all for older adults afflicted by memory disturbance.

In the current study we aimed to explore the potential usefulness of the DOP as a technique for enhancing the learning and recall of medical prescriptions in older adults. Therefore, we used a discriminative learning task where patients had to learn to associate a specific drug (a pill) with the time of day when the drug was supposed to be taken (morning, midday, or evening). To assess the effect of the DOP on the long-term memory, two memory tests were conducted, 1 h and 1 w after the learning phase. Based on the above-mentioned previous studies, our hypothesis is that the application of differential outcomes will significantly improve the learning and memory of the previously learned associations, compared to when non-differential outcomes were applied.

## Materials and Methods

### Participants

Two groups of participants volunteered to participate in this study. Participants in the younger group (12 females and 6 males; aged 19–34, mean age: 22.4 years, *SD*: 3.7) were 18 undergraduate students from the University of Almería (Spain), who received course credits for their participation. Participants in the older group (15 females and 3 males; aged 60–84, mean age: 71.9 years, *SD*: 6.7) were 18 healthy older adults from the Universidad Popular (Valencia, Spain).

All participants had normal or corrected-to-normal vision and they were free of medical conditions that could impair cognitive functioning or interfere with measures to be studied. The older adults did not present signs of dementia or cognitive impairment as assessed by the Mini-Mental State Examination (Folstein et al., 1975). Finally, the study was conducted in accordance with the ethical standars in the Declaration of Helsinki and was approved by the University of Almería Human Research Ethics Committee. Written informed consent was obtained from all participants.

### Stimuli and Apparatus

The stimuli consisted of three pictures of different pills (a blue, a pink, or a yellow pill; the sample stimuli) and three pictures of different times of day (early morning, midday, and night; the comparison stimuli). They were displayed on a white background on a color screen (15-inch VGA monitor) of an IBM-compatible computer.

Three pictures with positive emotional valence (a baby, a smiling woman, and a dog) along with the text “You may win a” followed by the name of a primary reinforcer (a book, a table game, or a music CD), were used as immediate secondary reinforcers (the outcomes). As in previous studies, participants were told that the more accurate they were on their response, the more probabilities to win one of the prizes they would have (e.g., Miller et al., 2002; López-Crespo et al., 2009; Plaza et al., 2011; Molina et al., 2015). Primary reinforcers were raffled off at the end of the study; all participants received one of the prizes for their participation.

E-prime software (Psychology Software Tools, 2012) was used to design the experimental task as well as to control the stimulus presentation and record participants’ responses.

### Procedure

Each participant was tested individually in a quiet room. The experiment consisted of two phases, the learning or training phase – which lasted for approximately 20 min – where participants performed a delayed conditional discrimination task and the memory phase – which lasted approximately 3 min – where participants were scheduled for two memory tests, one taking place 1 h later and the other 1 week following the training phase.

**FIGURE 1:**
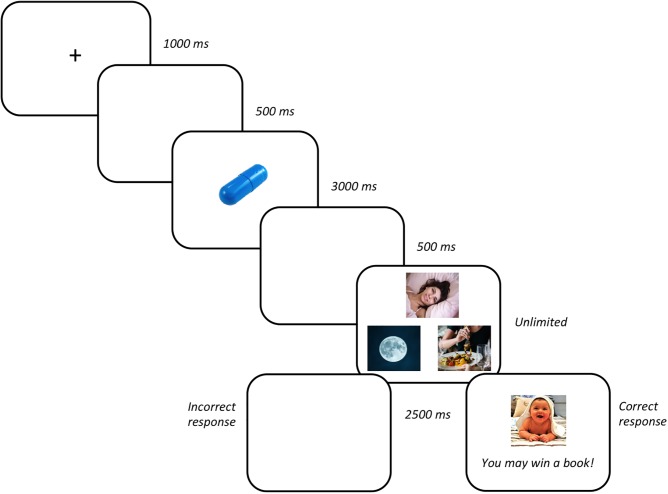
Stimuli sequence (from left to right).

In the learning phase, participants were instructed verbally while a sample trial was shown on the screen, to guess which pill (sample stimulus) was associated with each time of day and then to remember these associations. The experiment consisted of 54 trials grouped in three blocks of 18 trials each. The trial sequence (see **Figure 1**) began with a fixation cross presented for 1000 ms. The cross was replaced by a white screen for 500 ms and then the picture of a pill (sample stimuli) was displayed for 3000 ms. Following a delay of 500 ms during which the screen was blank, three pictures of the different times of day appeared until the participant responded by touching the screen. Following a correct response, participants received a secondary reinforcer (both a picture and a text indicating the prize – the primary reinforcer – they would win) for 2500 ms. Incorrect responses were followed by a blank screen for the same time (time-out period) as the outcome presentation.

All participants were randomly assigned to one of the two experimental training conditions. In the DOP condition, each sample stimulus was always associated with one specific outcome and correct responses to a particular stimulus led only to its associated outcome. For instance, the blue pill/early morning association was reinforced with the picture of a baby and the text “You may win a book!”; the pink pill/midday association with the picture of the smiling woman and the text “You may win a table game!”; and the yellow pill/night association with the picture of the dog and the text “You may win a music CD!” In the non-differential outcomes (NOP) condition, correct responses were reinforced with one of the randomly administered outcomes. The next trial started immediately after the reinforcer or time-out period.

In the memory phase, participants performed two recognition memory tests, one taking place 1 h after the training phase and the other 1 week later. The task consisted of three trials – one for each trained pill/time of day association. The stimulus sequence was identical to the learning phase with the exception that participant’s responses were not followed by any outcome. Participants were not informed in advance that a memory tests would be conducted after the learning phase.

### Statistical Analyses

For the learning phase, correct responses were grouped in six blocks of nine trials each and were submitted to a mixed ANOVA with Outcomes (DOP and NOP) and Age-group (younger adults and older adults) as the between-participants factor and Blocks of trials (1, 2, 3, 4, 5, and 6) as the within-participants factor. Median correct response times were submitted to a mixed ANOVA with Outcomes (DOP and NOP) and Age-group (younger adults and older adults) as the between-participants factor.

Finally, for the memory phase, percentages of correct responses from the two memory tests were also submitted to a mixed ANOVA with Outcomes (DOP and NOP) as the between-participants factor and Test time (1 h and 1 week) as the within-participants factor.

The significance level was set at *p* ≤ 0.05.

## Results

### Learning Phase

#### Accuracy Analysis

**FIGURE 2:**
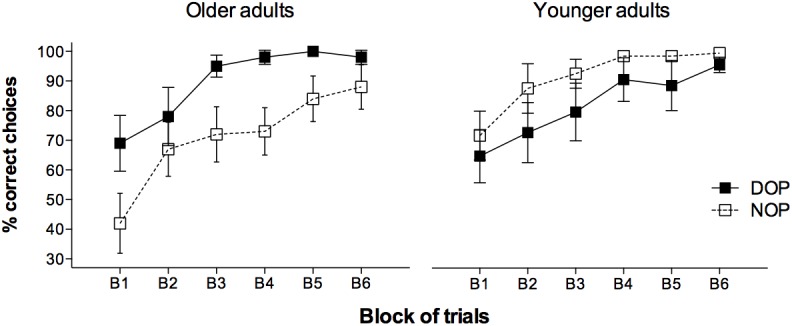
Mean percentages of correct responses for the younger and older adults groups in the learning phase as a function of Blocks of trials (six blocks of nine trials each) and Outcomes (DOP and NOP). Error bars show the standard error of the mean.

The analysis conducted on percentages of correct responses (see **Figure 2**) showed a significant main effect of Blocks [*F*(5,160) = 22.22, *p* < 0.001, $\eta_{p}^{2}$ = 0.410] due to accuracy linearly increased with blocks of trials (62, 76, 85, 90, 93, and 95% correct in blocks 1, 2, 3, 4, 5, and 6, respectively). The Outcomes × Age-group interaction reached statistical significance [*F*(1,32) = 6.13, *p* = 0.019, $\eta_{p}^{2}$ = 0.161]. The analysis of the interaction revealed that the difference between the differential outcomes and non-differential outcomes conditions was only observed in the older group [*F*(1,16) = 4.70, *p* = 0.046, $\eta_{p}^{2}$ = 0.227] but not in the younger group [*F*(1,16) = 1.60, *p* = 0.224, $\eta_{p}^{2}$ = 0.091], indicating that only the older adults performed significantly better on the task when differential outcomes were arranged (89% vs. 71% accuracy in the differential and non-differential conditions, respectively). Importantly, the older adults’ performance did not differ from that of the younger adults in the DOP condition [*F*(1,16) = 0.98, *p* = 0.337, $\eta_{p}^{2}$ = 0.058].

Although the three-ways Outcomes × Age-group × Block interaction did not prove statistically significant [*F*(5,160) = 0.58, *p* = 0.717, $\eta_{p}^{2}$ = 0.018], it is worth noting that older adults benefited from training with the differential outcomes since the first block of trials (69% vs. 42% correct, respectively), being the difference marginally significant [*F*(1,16) = 3.90, *p* = 0.066, $\eta_{p}^{2}$ = 0.196]. However, the differential outcomes benefit was not apparent in the younger adults in this first block of trials (65% vs. 72% correct) [*F*(1,16) = 0.265, *p* = 0.614, $\eta_{p}^{2}$ = 0.016]. Importantly, older adults assigned to both conditions showed a similar accuracy when the first two trials were analyzed [*F*(1,16) = 2.44, *p* = 0.138, $\eta_{p}^{2}$ = 0.132] indicating that they all were naive to the task at the beginning of the training phase.

### Reaction Times Analysis

The analysis conducted on median correct reaction times (RTs) showed a significant main effect of Age-group [*F*(1,32) = 4.53, *p* = 0.041, $\eta_{p}^{2}$ = 0.124], that is, in general, younger adults were faster than older adults (1323 ms vs. 1700 ms, respectively). The Outcomes × Age-group interaction was also significant [*F*(1,32) = 4.76, *p* = 0.037, $\eta_{p}^{2}$ = 0.130]. The analysis of the interaction revealed a significant effect of Outcomes only in the older adults group [*F*(1,16) = 4.95, *p* = 0.041, $\eta_{p}^{2}$ = 0.236] who showed faster RTs when differential outcomes were arranged (1355 ms vs. 2046 ms in the differential and non-differential condition, respectively). Importantly, additional analysis revealed that RTs were longer for older adults than for younger adults in the NOP condition [*F*(1,16) = 5.46, *p* = 0.033, $\eta_{p}^{2}$ = 0.254], but not in the DOP condition [*F*(1,16) = 0.005, *p* = 0.946, $\eta_{p}^{2}$ = 0.000]. No other effects, nor their interaction, reached statistical significance (*p*s > 0.05).

### Memory Phase

**FIGURE 3:**
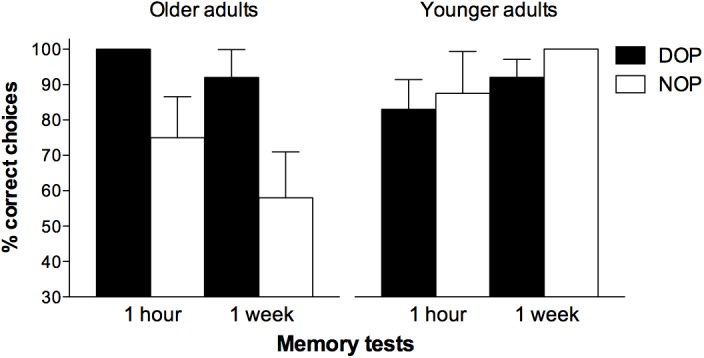
Mean percentages of correct responses obtained by participants in the memory recognition tests as a function of Outcomes (DOP and NOP) and Time of testing (1 h and 1 week). Error bars show the standard error of the mean.

**Figure 3** shows the percentage of correct responses obtained by participants in the memory tests. The analysis of accuracy data revealed a significant Age-group × Test time interaction [*F*(1,28) = 5.07, *p* = 0.032, $\eta_{p}^{2}$ = 0.153] due to participants in both groups showing differential performance in the 1-week test [*F*(1,30) = 5.03, *p* = 0.032, $\eta_{p}^{2}$ = 0.144] but not in the 1 h test [*F*(1,30) = 0.04, *p* = 0.844, $\eta_{p}^{2}$ = 0.001]. The Outcomes × Age group interaction was also significant [*F*(1,28) = 5.37, *p* = 0.028, $\eta_{p}^{2}$ = 0.161]. Further analysis of the interaction revealed a significant effect of Outcomes in the older adults group [*F*(1,14) = 5.79, *p* = 0.031, $\eta_{p}^{2}$ = 0.293] but not in the younger group [*F*(1,14) = 0.45, *p* = 0.515, $\eta_{p}^{2}$ = 0.031], indicating that only older adults performed significantly better on the two memory task when differential outcomes were arranged (96% vs. 67% correct in the differential and non-differential conditions, respectively). It is worth noting that additional analyses revealed that although the long-term retention of the learned information was better for the younger adults than for the older adults in the NOP condition (94% vs. 67%) [*F*(1,14) = 4.36, *p* = 0.055, $\eta_{p}^{2}$ = 0.238], it was similar for both groups in the DOP condition (88% vs. 96%) [*F*(1,14) = 1.06, *p* = 0.321, $\eta_{p}^{2}$ = 0.070]. No other effects, nor their interaction, reached statistical significance (*p*s > 0.05).

## Discussion

The present study was designed to explore whether the use of the DOP, a procedure imported from animal studies, might improve the learning and long-term retention of pill/time of day associations in older adults. Memory for this type of medical treatment-related information is a prerequisite for a good adherence to treatment, especially for those who have multiple morbidities or chronic illnesses and must take several medications (Kripalani et al., 2007; Tavares et al., 2013), as it is common for older adults. In fact, one important reason for this population to fail to correctly handle critical aspects related to their diseases is that they forget to take their medications as prescribed (Jimmy and Jose, 2011). Thus, it is of crucial relevance to design appropriate interventions that help patients minimize their memory loss concerning medical treatment. The present results suggest that the DOP might be one of such therapeutic techniques. As expected, participants showed better performance (higher accuracy, in both learning and memory phases, and faster RTs) when differential outcomes were used compared to when the same outcomes were randomly administered. Also, older adults’ memory under the DOP resisted better the time pass. As far as we know, this is the first demonstration that the DOP is an effective technique to enhance older adults’ learning and retention at both the short and long-term, of medical treatment-related information. It is also worth noting that participants in the DOP group showed equivalent performance to younger adults in both learning and memory phases. Younger adults did not take any advantage of the DOP, probably because the task used in the present study was too easy to perform for them. Similar results were reported in previous studies (Estévez et al., 2001, 2016; Plaza et al., 2011, 2012, 2013), indicating a modulation of the beneficial effects of applying differential outcomes on learning and memory by task difficulty. Furthermore, it has also been observed that when participants find the task rather easy to perform, they usually show overall higher performance in the NOP compared with the DOP, although in the present experiment this difference is not statistically significant^1^ (see, for example, Esteban et al., 2014b, Figure 2, 7-years-old group; Martínez et al., 2012, Figure 4, control group).

These results also extend those obtained by Molina et al. (2015) in healthy young adults who had to learn and retain in their memory, the pills that were associated with specific disorders. They found that the use of differential outcomes following their correct responses produced better discriminative learning and long-term memory of the previously learned pill/disorder associations. The ecological nature of the task used in both Molina et al.’s (2015) and the present study, allows us to highlight the potential of the DOP as a useful technique for intervention programs targeted at increasing the learning and recall of crucial medical treatment-related information in different populations, especially in those with memory impairments. However, although these findings are very promising, further studies should be conducted to test whether enhancement in the learning and retention of this kind of information (e.g., knowing the time when each drug is supposed to be taken) observed when the DOP is applied, might foster patients’ adherence to medical prescription and subsequent health improvement in everyday life. In futher studies a larger sample size as well as a more balanced gender distribution of the participants than that used in the present research would be desirable to increase the representativeness and generalization of the results.

The two-memory system model suggested by Savage et al. (1999), Savage (2001), Savage and Ramos (2009), an extension of the expectancy theory (Trapold and Overmier, 1972), can explain the benefits of using the DOP in a variety of learning and memory tasks, including the present one. The model proposes the existence of two different memory systems that are activated by differential and non-differential outcomes. Under non-differential outcomes conditions the only source of information to solve the task is the retrospective recall of the sample stimulus (the pill), which it is based on an explicit cholinergic-dependent memory system (retrospective memory) that is rather compromised in the elderly (Hedden and Gabrieli, 2004), above all when working memory demands are high. Therefore, it is not surprising that older people showed more difficulties to perform the task used in the present study than younger people under the NOP condition. In contrast, when the DOP is used, the continuous pairing of a specific stimulus (the pill) with a unique outcome, results in the activation of specific stimulus-outcome expectations. Expectations can be thought of as prospective memory representations elicitated by the sample stimuli (the pills) of which outcomes will be forthcoming. As learning takes place, these expectations become stronger, providing an additional source of information that improves accuracy and overcome working memory demands. Therefore, an implicit glutamatergic-dependent memory system (prospective memory) is thought to be involved in the DOP. According to this perspective, those populations in which the cholinergic system and/or explicit memory is deteriorated, as in normal and pathological aging (Hedden and Gabrieli, 2004; Schliebs and Arendt, 2006), still can take advantage of the differential outcomes methodology because it just requires the correct functioning of a relatively well-preserved system, the prospective memory. Results from different studies support the two-memory system model, includying a research using functional MRI. Mok et al. (2009) found with humans that different brain regions are recruited when differential and non-differential outcomes are arranged; especifically, they observed greater hippocampal activation under non-differential and greater activation of the angular gyrus of the posterior parietal cortex under differential outcomes. The former is known to be involved in retrospective memory (e.g., Savage et al., 2004) whereas the latter seems to be involved in prospective memory.

## Conclusion

The current research demonstrates the usefulness of the DOP as an appropriate technique to enhance elderly people’s learning and long-term retention of medical treatment-related information. Future studies should investigate the potential impact of this improvement on their adherence to treatment in their daily life as well as its effectiveness in other age-related diseases such as mild cognitive impairment, dementia, or other neurodegenerative disorders, at least in early stages of the disease. If a better adherence to treatment were found, this might have a great impact on their daily functioning by improving their autonomy and quality of life, but also in reducing the high costs associated with the public health system.

## Author Contributions

VP was responsible for writing the manuscript and contributed to the design of the study and statistical analysis. MM was responsible for data collection and participated in statistical analysis. LJF contributed to data interpretation and assisted with writing the manuscript. AFE was responsible for the design of the study and statistical analysis, contributed to data interpretation, and assisted with writing the manuscript. All authors approved the final version of the manuscript for submission.

## Conflict of Interest Statement

The authors declare that the research was conducted in the absence of any commercial or financial relationships that could be construed as a potential conflict of interest.

## Footnotes

1 In further analyses (results not shown), we were able to discount the influence of possible outliers biasing the results of the younger adults group in the NOP condition.
